# The Psychological Risks Associated With the Non-medical Switch From Biologics to Biosimilars

**DOI:** 10.3389/fpsyg.2021.605643

**Published:** 2021-01-27

**Authors:** Davide Mazzoni, Claudia Vener, Ketti Mazzocco, Dario Monzani, Gabriella Pravettoni

**Affiliations:** ^1^Department of Oncology and Hemato-Oncology, University of Milan, Milan, Italy; ^2^Applied Research Division for Cognitive and Psychological Science, European Institute of Oncology IRCCS, Milan, Italy

**Keywords:** nocebo effect, shared decision-making, adherence, rheumatology, oncology

## Introduction

Biological products are therapeutic agents produced using a living system or organism. In many cases, access to these products is limited due to their expensive cost (Chow et al., 2011). A biosimilar is a biological product that is highly similar (not identic) to, and has no clinically meaningful differences from, an existing reference biological product on the market (Desai et al., 2020). “Non-medical” switching is the switching of a patient's medicine for reasons other than the patient's health and safety, like the reduction of costs (Dolinar et al., 2019).

The therapeutic interchangeability and substitution of a biologic with the biosimilar product have recently increased its relevance for the scientific and political debate in Italy as in other European countries (Kurki et al., 2017; La Noce and Ernst, 2018; Dolinar et al., 2019). In Europe, substitution of a reference medicine with a biosimilar product is encouraged for treatment-naïve patients. However, a quite cautious approach has been taken with regard to switching patients on the reference product to a biosimilar product, with differences across countries (La Noce and Ernst, 2018). In Italy, such debate was alimented, among the others, by the position papers from the Italian Medicines Agency (AIFA) and several medical associations in the areas of rheumatology, gastroenterology, oncology, and nephrology (Brogonzoli et al., 2018; ADOI, 2019).

One of the core issues of this debate is represented by the switch to a biosimilar in patients being treated with the reference biologic. As biosimilars are interchangeable, they can be expected to produce the same clinical result as the reference product in any given patient and, for a biologic product that is administered more than once, that the risk of alternating or switching between use of the biosimilar product and the reference product is not greater than the risk of maintaining the patient on the reference product (FDA, 2019).

In 2018, for the first time, the AIFA report, starting from the demonstrated risk-benefit equality between the biosimilar drug and the reference originator drug, considered biosimilars as interchangeable with the corresponding reference originators (even living to the clinician the final decision and excluding the automatic shift) (Brogonzoli et al., 2018). In this regard, many Italian medical organizations, although agreeing on their use in primary naïve patients, further remarked some concerns on the switch in patients with chronic or severe diseases, given that stable medication regimens are essential to effective disease management (ADOI, 2019; Dolinar et al., 2019).

We cannot summarize here such complex debate, which includes contributions based on economic and clinical factors, ranging from the biosimilars' productive process to the constitutional rights (Kurki et al., 2017; Dolinar et al., 2019). The present contribution, even if embedded in this debate, focuses on the (less debated) risks related to the patients' perspective about the switch. Building on a narrative literature review, the specific expertise of our research unit in the psychological processes implicated by biologic treatments, and the recent positions expressed by patients' organizations, in the next paragraphs we will review some of the potential risks connected with the switch in some interrelated domains: expectations, perceived drug efficacy, patients' adherence, and shared decision making. We close our contribution discussing the implications for clinical practice and public health, proposing some strategies to mitigate these potential risks in switching from a reference medicine to a biosimilar.

## Methods

The databases PubMed, Web of Science, and PsycInfo were searched using the two terms “biologic(al)(s)” and “biosimilar(s)” together in article title, or abstract, or subject. The search was limited to articles that were published in the last 10 years (2011–2020) in English. The search provided, respectively: 643 articles in PubMed, 909 in Web of Science, and 3 articles in PsycInfo. Only the articles focusing on the non-medical switch from biologics to biosimilars were thus considered. Additional literature was obtained through searching references in the manuscripts (snowball method).

The full texts of the relevant articles (see the references) were collected and analyzed in order to identify the main potential risks for patients, related to the switch from a biologic to a biosimilar product. The recent positions expressed by patients' organizations were obtained combining the consultation of documents obtained through web searches and phone interviews with patients' organizations representatives. Two representatives of the patients' organizations that recently took an explicit position on the topic were contacted (Patients' Organizations, 2019). Interviews were performed as a form of integration and clarification of the position of patients' organizations that was expressed in the retrieved documents. The potential risks connected with the switch were thus grouped in three main domains: negative expectations, perceived drug efficacy and adherence, and shared decision making.

## Negative Expectations

One of the psychological issues related to the switch has to do with the negative expectations and the consequent discomfort with switching to a biosimilar product (Rezk and Pieper, 2017; Odinet et al., 2018). In this regard, we must consider that, despite the evidence supporting the similarities between biosimilars and the reference drugs, some Italian and European surveys have indicated that prescribers do not have a strong understanding of the complex manufacturing process, approval requirements, or ongoing regulation of biologic and biosimilar products (Pasina et al., 2016; Cohen et al., 2017; O'Callaghan et al., 2017). In this sense, specialists who are more uncertain about their comparability in safety and efficacy may have reservations and be cautious in adopting biosimilars (Cohen et al., 2017).

In turn, the prescribers' lack of knowledge and skepticism could negatively influence the patients' expectations. This seems consistent with some surveys on patients from different European countries, which found also some skepticism about “non-medical” switch (Jacobs et al., 2016; Dolinar et al., 2019). Moreover, in the last years, many Italian patients' organizations felt the need to express their point of view in separate and joint documents, in which they remarked the importance of shared decision making and the importance of an individualized approach (ANMAR, 2018). In summary, it is likely that this knowledge gap, both in doctors and patients, could contribute to a background scenario of negative expectations (Jacobs et al., 2016; Pasina et al., 2016; Cohen et al., 2017; O'Callaghan et al., 2017).

## Perceived Drug Efficacy and Adherence

Another potential risk about the switch is about the negative impact that the above-mentioned negative expectations could have in terms of perceived efficacy and therapeutic adherence. These outcomes are strictly connected with the placebo and nocebo effects, which are carefully considered in trials but less often considered for their potential effects in clinical practice (Barsky et al., 2002; Rezk and Pieper, 2017; Odinet et al., 2018). In simple terms, according to the nocebo effect, if patients anticipate a negative effect associated with a medication or change in medication, they may then experience negative side effects or worsening of symptoms. The implications are that the nocebo effect can balance or limit drug outcomes and decrease the patient's therapeutic adherence. Similarly, negative patients' expectations on the switch from the biologic to the biosimilar could, at least in theory, produce a negative effect in terms of efficacy and lead to non-adherence (Petrie and Rief, 2019). A recent systematic literature review showed that current evidence is insufficient to confirm a biosimilar nocebo effect, although higher discontinuation rates in some studies support this theory (Odinet et al., 2018). This discontinuation could manifest quite suddenly or do not emerge until an adverse event will be attributed more easily by the patient to the new drug.

## Shared Decision-Making

Finally, a less debated challenge is represented by the way through which the decision to switch should be communicated and negotiated with the patients. Shared decision making is a process by which patients and physicians discuss and evaluate the alternatives for a medical decision together (Gorini and Pravettoni, 2011; Marzorati and Pravettoni, 2017). The first step consists of making the patient aware of the diagnostic and treatment pathways (e.g., risks and benefits associated with each available alternative), while the second one consists of asking the patient to communicate preferences and concerns about the different alternatives.

Despite the shared decision-making models suggest that the patients should be adequately informed about the new therapies, this could result quite difficult because of the complexity of the topic. Moreover, some of the few authors who recently investigated the impact of shared decision-making, concluded that following a shared decision-making approach in the transition may lead to even lower retention rates than those observed using a more directive approach (Müskens et al., 2020).

In any case, according to many patients' organizations, the choice about the possible switch should be taken by the doctor in agreement with the patient (ANMAR, 2018). Building on our clinical practice, we believe that specific attention should be given to the process that leads to this agreement, considering all the patient's resistances. For example, receiving benefits from the current treatment with a reference product, a lack of understanding about the nature and efficacy of biosimilars, the absence of perceived personal advantages related to the switch, may all inhibit the willingness to agree on the switch.

## Discussion

For all these reasons, the identification of possible strategies to mitigate the risks represents both an important challenge and an interesting line of research for the future. To our knowledge, it is possible to identify at least three key strategies that have an impact on the above-mentioned risks (Kristensen et al., 2018). The first strategy consists in increasing patient and healthcare professionals' understanding of biosimilars. For example, informing patients about safety issues would reduce the negative expectations (as they largely depend on the lack of understanding of biologic and biosimilar products) and the consequent nocebo effect, facilitating the first step of the shared decision-making process (i.e., making the patient aware, ensuring shared decision making). As regards healthcare professionals, there is a need for evidence-based education about biosimilars across specialties, reducing the knowledge gaps on their definition and approval process (Cohen et al., 2017).

The second strategy consists in the adoption of a positive framing. This means that for effective physician-patient communication, the equality of the treatments as assessed by independent regulators should be stressed, instead of overemphasizing the remote chance of a small difference with unknown clinical consequences. This would prevent physicians from instilling negative expectations in patients, reducing the nocebo effect and facilitating the second step of the shared decision-making process (i.e., patients' expression of preferences about the different alternatives). Finally, the third strategy consists in the use of a planned and managed switching program guidance. This means that all the healthcare professionals who are involved in the patients' management speak the same language, ensuring that no divergent opinions are being expressed to patients regarding the agreed treatment strategies. This includes also the essential nurses' collaboration (Armuzzi et al., 2019).

Moreover, these strategies (because of their positive effect on expectancies, perceived efficacy, adherence, and shared decision-making) may have strong implications for public health and clinical practice. In fact, from a public health perspective, dealing with nocebo effects could result in additional patient clinic visits, which places more pressure on a healthcare system and reduces the cost savings of biosimilars (Kristensen et al., 2018). In the clinical practice, the patients' doubts, if not adequately recognized and addressed could ultimately generate worse outcomes. In this sense, each patients' psychological characteristics (such as anxiety, depression, and the tendency to somatize) and his/her perspective on the issues of therapeutic substitution should be considered for reducing the risks for health and related costs.

## Limitations

A limitation of the present work is that it is mostly based on a narrative rather than systematic review. With the increase of the studies on the topic, future comprehensive and systematic reviews will ensure to consider all the relevant papers on the topic. Moreover, in the future the patients' perspectives that we considered through the lenses of our specific expertise and the recent positions expressed by patients' organizations, could be in-depth investigated through the integration of quantitative and qualitative methods. Nevertheless, the positions expressed in this paper are fully referenced and strongly related with patients' and health professionals' experiences.

## Author Contributions

DMa wrote the first draft of the manuscript. CV wrote sections of the manuscript. KM, DMo, and GP contributed to the manuscript revision, read, and approved the submitted version. All authors contributed to the article and approved the submitted version.

## Conflict of Interest

The authors declare that the research was conducted in the absence of any commercial or financial relationships that could be construed as a potential conflict of interest.
